# Evaluation and implications of natural product use in preoperative patients: a retrospective review

**DOI:** 10.1186/1472-6882-9-38

**Published:** 2009-10-13

**Authors:** Allison R King, Flint S Russett, Joyce A Generali, Dennis W Grauer

**Affiliations:** 1 University of Kansas Drug Information Center, 3901 Rainbow Boulevard, MS 4040, Kansas City, Kansas, 66160, USA; 2 St. Claire Regional Medical Center, Department of Pharmacy, 222 Medical Circle, Morehead, Kentucky, 40351, USA; 3 Drug Information Center, University of Kansas, 3901 Rainbow Boulevard, MS 4040, Kansas City, Kansas, 66160, USA; 4 Department of Pharmacy Practice, Malott hall, Room 2058, 1251 Wescoe Hall Drive, Lawrence, Kansas 66045-7582, USA; 5 University of Kansas Hospital, 3901 Rainbow Boulevard, MS 4040, Kansas City, Kansas, 66160, USA

## Abstract

**Background:**

Medication Reconciliation and Medication Safety are two themes emphasized in a variety of healthcare organizations. As a result, health care facilities have established methods for obtaining a patient's medication history. However, these methods may vary among institutions or even among the health care professionals in a single institution, and studies have shown that patients are reluctant to disclose their complementary and alternative medicine use to any health care professional. This lack of disclosure is important in surgical patients because of potential herbal interactions with medications and drugs used during the surgical procedure; and the potential for adverse reactions including effects on coagulation, blood pressure, sedation, electrolytes or diuresis. Therefore, the objectives of this study are to identify patterns of natural product use, to identify potential complications among patients scheduled for surgery, to improve existing medication reconciliation efforts, and to develop discontinuation guidelines for the use of these products prior to surgery.

**Methods:**

A retrospective review of surgery patients presenting to the Anesthesia Preoperative Evaluation Clinic (APEC) at the University of Kansas Hospital was conducted to identify the prevalence of natural product use. The following data was collected: patient age; gender; allergy information; date of medication history; number of days prior to surgery; source of medication history; credentials of person obtaining the history; number and name of prescription medications, over-the-counter medications and natural products; and natural product dosage. Following the collection of data and analysis of the most common natural products used, possible complications and interactions were identified, and a protocol regarding the pre-operative use of natural products was developed and implemented.

**Results:**

Approximately one-fourth of patients seen in the APEC indicated the use of natural products. Patients taking natural products were significantly older, were more likely to undergo cardiac or chest surgery, and were more likely to be taking more prescription and non-prescription medications (all p < 0.001).

**Conclusion:**

Based on the results of this study, it is concluded that there is a need for established guidelines regarding discontinuation of selected natural products prior to surgery and further education is needed concerning the perioperative implications of natural products.

## Background

Complementary and alternative medicine (CAM), defined as "a group of diverse medical and health care systems, practices, and products that are not presently considered to be part of conventional medicine", consists of a wide variety of healing practices including acupuncture, chiropractic therapy, traditional Chinese medicine, massage, homeopathic medicine, and dietary supplements [1]. A dietary supplement is further defined as "a product (other than tobacco) taken by mouth that contains a 'dietary ingredient' intended to supplement the diet". This includes vitamins, minerals, herbs or other botanicals, amino acids, enzymes, organ tissues, and metabolites [1]. Use of CAM has greatly increased over the past decade. A general population survey conducted in 1990 and then again in 1997 showed that overall CAM use in the United States had increased from 33.8% in 1990 to 42.1% in 1997 (p < 0.001). In the same survey, herbal use had also dramatically increased from 2.5% in 1990 to 12.1% in 1997 (p < 0.001) [2]. More recent studies have shown that herbal or natural product use is even more prevalent in certain US population subsets: elderly adults with anxiety or depression (17.3%); adults presenting to ambulatory care clinics (17-37%); African Americans (19.6%); adult residents of North Carolina (19.9%); diabetic adults (22.3%); community dwelling elderly adults (20.7%); surgical patients (37.5%); Hispanic California residents (57%); elderly California residents with chronic illnesses (53%); and Hispanic surgical patients (61.7%) [3-12].

Medication Reconciliation and Medication Safety are two themes emphasized in a variety of healthcare organizations (e.g., Health-System Pharmacy 2015 Initiative, Joint Commission on Accreditation of Healthcare Organizations, Institute for Healthcare Improvement, and the National Quality Forum). As a result, health care facilities have established methods for obtaining a patient's medication history. Unfortunately, these methods may vary among institutions or even among the health care professionals obtaining the medication history in a single institution. In the previously mentioned studies, responders were specifically asked about their CAM or natural product use. However, other studies have shown that CAM disclosure rates vary from 30% to 46.7% [3-13]. There are various reasons why disclosure rates are so low including: patients are never specifically asked about CAM or natural product use, embarrassment, or the belief that herbal products are not medicine [13,14]. Additionally, environment may also impact the patient's viewpoint of safety. If a patient does not feel safe or is fearful of provider judgment, they may be less likely to disclose CAM or natural product use.

There are various reasons why people may not consider herbal products medicine, such as low cost, availability without a prescription, and because they are marketed as all-natural products [13,14]. Patients may associate "all-natural" with safe; however, they may not be aware that many potentially toxic prescription medications, including chemotherapeutic agents, are derived from plants, e.g. digitalis (foxglove plant), quinine (cinchona bark), vincristine (periwinkle), and paclitaxel (Pacific yew tree) [13,15].

While it is always important for health care professionals to be fully aware of all medicines or therapies a patient is taking, it is especially important in surgical patients because of potential herbal interactions with medications and anesthetic agents used during the surgical procedure; and the potential for adverse reactions including effects on coagulation, blood pressure, sedation, electrolytes or dieresis [9,14-22]. Patients having elective surgery are usually counseled to discontinue certain medications prior to surgery, especially anticoagulation agents, diuretics, and oral hypoglycemics. It is important for surgeons and anesthesiologists to be familiar with natural products and their perioperative implications. However, one survey demonstrated that the majority of physicians were not familiar with the side effect profiles of many herbal products and did not recommend stopping herbal medications in the perioperative period [15]. Another study evaluating the knowledge and practice of family medical residents found that, when asked to match an herbal product to a drug interaction, the mean test score was 32% [23].

## Methods

Pharmacist-mediated medication reconciliation efforts at the University of Kansas Hospital (KUH) have documented preoperative use of natural products by surgical patients. The Anesthesia Preoperative Evaluation Clinic (APEC) is a clinic within KUH where patients scheduled for surgery receive a pre-surgery evaluation by a nurse and meet with a pharmacist to discuss their medications. KUH has developed a Pre-Surgical Medication Counseling Form which provides discontinuation counseling information for specified medications. The role of the pharmacist is to obtain an accurate medication history from the patient and provide recommendations for discontinuation of the patient's medications in regards to their surgical procedure. The role of the nurse is to obtain an accurate medical history from the patient and draw any necessary laboratory work and conduct an EKG, if necessary. If any EKG abnormalities are noted, or if concerns are raised about the patient's safety during surgery, an anesthesiologist will meet with the patient and address the concerns. Most patient appointments in the APEC are scheduled one week prior to surgery; however walk-in appointments are welcome.

Current recommendations given to patients regarding natural product use include a statement recommending discontinuation 14 days prior to surgery. This implicates a potential problem: as noted above, most patients are scheduled one week prior to their surgery. Additionally, two of the three patient rooms had a chart which included pharmacological effects, perioperative concerns and preoperative discontinuation of specific herbs (see Table 1). However, the chart was not complete or accurate and did not provide references. In consideration of these current problems, the primary objective of this study is to identify patterns of natural product use and their potential complications among patients scheduled for surgery. Secondary objectives include improving existing medication reconciliation efforts, and developing discontinuation guidelines for the use of these products prior to surgery.

**Table 1:** Original Chart in APEC

**Herb**	**Pharmacological Effect**	**Perioperative Concerns**	**Preoperative Discontinuation**
Echinacea	Activation of cell-mediated immunity	Allergic reactions, decreased effectiveness of immunosuppressants; potential for immunosuppression with long-term use	No data

Ephedra (ma huang)	Increased HR and BP through direct and indirect sympathomimetic effects	Risk of MI and stroke from tachycardia and htn; ventricular arrhythmias with halothane; long-term uses depletes endogenous catecholamines and may cause intraoperative hemodynamic instability; life-threatening interaction with MAOI's	At least 24 hours before surgery

Garlic	Inhibition of platelet aggregation, increased fibrinolysis, equivocal antihypertensive activity	Potential to increase risk of bleeding, especially when combined with other medications that inhibit platelet aggregation	At least 7 days before surgery

Ginkgo	Inhibition of platelet-activating factor	Potential to increase risk of bleeding, especially when combined with other medications that inhibit platelet aggregation	At least 36 hours before surgery

Ginseng	Lowers blood glucose; inhibition of platelet aggregation; increased PT-PTT in animals; varied others	Hypoglycemia; potential to increase risk of bleeding; potential to decrease anticoagulation effects of warfarin	At least 7 days before surgery

Kava	Sedation, anxiolysis	Potential to increase sedative effect of anesthetics; potential for addiction, tolerance, and withdrawal after abstinence unstudied	At least 24 hours before surgery

St. John's Wort	Inhibition of neurotransmitter reuptake, MAO inhibition unlikely	Induction of P450 enzymes (CYP 3A4) affecting cyclosporine, warfarin, steroids, protease inhibitors, and possibly benzo's, calcium channel blockers, and many other drugs; decreased serum digoxin levels	At least 5 days before surgery

Valerian			

A retrospective review of surgery patients presenting to the Anesthesia Preoperative Evaluation Clinic (APEC) was conducted to identify the prevalence of natural product use. The following data was collected: patient age; gender; allergy information; date of medication history; number of days prior to surgery; source of medication history; credentials of person obtaining the history; number and name of prescription medications, over-the-counter (OTC) medications and natural products used; and dosages of natural products. Following collection of data and determination of the most common natural products used, possible complications and interactions were identified. A protocol regarding the pre-operative use of natural products was developed and is in the process of being implemented.

Consultation with our institution's ethics committee was not required as our study was an observational retrospective review. Furthermore, as consultation did not occur, a waiver was not received. Statistical analyses were conducted by an independent, outside party. Descriptive analysis of the data was performed. T-tests were used to compare continuous variables and Chi-square tests used for categorical variables. Alpha was set at 0.05.

## Results

Between July 1, 2006 and August 14, 2006, a total of 434 patients presented to APEC, of which 104 reported current natural product use. Thus, 23% of the sample reported using natural products. Demographics of the patients are reported in Table 2.

**Table 2:** Demographics of surgical patients presenting to the Anesthesia Preoperative Evaluation Clinic (7/11/06 to 8/14/06)^1^

	**Total Number of patients (n = 434)**	**Patients Taking Natural Products (n = 104)**	**Patients Not Taking Natural Products (n = 330)**	**Statistical Significance^2^**
Mean Age (yrs)	54 ± 17	62 ± 12	51 ± 18	p < 0.001

Gender				NS (p = 0.239)

Male	187 (43)	50 (48)	137 (42)	

Female	247 (57)	54 (52)	193 (58)	

Allergies				NS (p = 0.192)

None	204 (47)	45 (43)	159 (48)	

Unknown	8 (2)	0 (0)	8 (2)	

Patients with Allergies	222 (51)	59 (57)	163 (19)	

Antibiotics	174 (40)	42 (40)	132 (40)	NS (p = 0.439)

Analgesia	100 (23)	30 (29)	70 (21)	NS (p = 0.107)

Other	97 (22)	26 (25)	71 (22)	NS (p = 0.457)

Surgical-associated	19 (4)	4 (4)	15 (5)	NS (p = 0.761)

Mean Time Before Surgery (days)	6.235 ± 7	6.22 ± 6	6.24 ± 7	NS (p = 0.9791)

Type of Surgery				

Genitourinary	130 (30)	28 (27)	102 (31)	NS (p = 0.439)

GI/Abdomen	71 (16)	12 (12)	59 (18)	NS (p = 0.127)

Joint	56 (13)	17 (16)	39 (12)	NS (p = 0.230)

Head, Eyes, Ears, Nose, Throat	47 (11)	9 (9)	38 (12)	NS (p = 0.413)

Spine	45 (10)	11 (11)	34 (10)	NS (p = 0.889)

Cardiac/Chest	41 (9)	18 (17)	23 (7)	p = 0.002

Breast	26 (6)	5 (5)	21 (6)	NS (p = 0.560)

Other/Unknown/Not Specified	14 (3)	4 (4)	10 (3)	NS (p = 0.682)

None	4 (1)	0 (0)	4 (1)	^3^

Interviewer				NS (p = 0.179)

Student	302 (70)	76 (73)	226 (68)	

Resident	16 (4)	6 (6)	10 (3)	

Pharmacist	116 (27)	22 (21)	94 (28)	

Source of History				NS (p = 0.394)

Patient	388 (89)	90 (87)	298 (90)	

Family	66 (15)	17 (16)	49 (15)	

Documentation	64 (15)	22 (21)	42 (13)	

Outside pharmacy	27 (6)	8 (8)	19 (6)	

Not specified	36 (8)	10 (10)	26 (8)	

Mean number of medications/natural products				

Prescription	5 ± 4	6 ± 4	5 ± 4	p < 0.001

Non-prescription	2 ± 2	3 ± 2	2 ± 2	p < 0.001

Natural Products	0.4 ± 1	1.9 ± 2	0	^3^

^1^Values are number (percentage) unless otherwise indicated

^2^T-tests used for to compare continuous variables and Chi-square tests used for categorical variables

^3^Data lacking power for statistical purposes

When evaluating the overall number of patients presenting to APEC, a majority were female (57%). Mean age was 54 years and most (53%) reported allergies. On average, patients presented to the clinic 6.235 ± 7 days before their surgery. Genitourinary was the most common type of surgery reported (30%); followed by GI/abdomen (16%); joint (13%); head, eyes, ears, nose, throat (11%); and spine (10%). Interviews were conducted mostly by pharmacy students (70%) with most information being reported by patients (89%). Other sources of history included family (15%), medication lists (15%), and outside pharmacies (6%). Patients reported taking 5 +/- 4 prescription medications, 2 +/- 2 non-prescription medications, and 0.4 +/- 1 natural products.

Compared to patients not taking natural products, patients taking natural products were significantly older (62 +/- 12 years of age versus 51 +/- 18 years of age, p < 0.001), and genitourinary was the most common type of surgery reported (27% and 31%, respectively, p = 0.239)). However, more patients taking natural products were scheduled for cardiac surgeries than patients not taking natural products (17% versus 7%, p = 0.002). Significantly higher prescription and non-prescription drug use was reported in patients taking natural products (6 +/- 4 and 3 +/-2, respectively, p < 0.001 for both) versus those not taking natural products (5 +/- 4 and 2 +/- 2, respectively) (p < 0.001).

**Figure 1:**
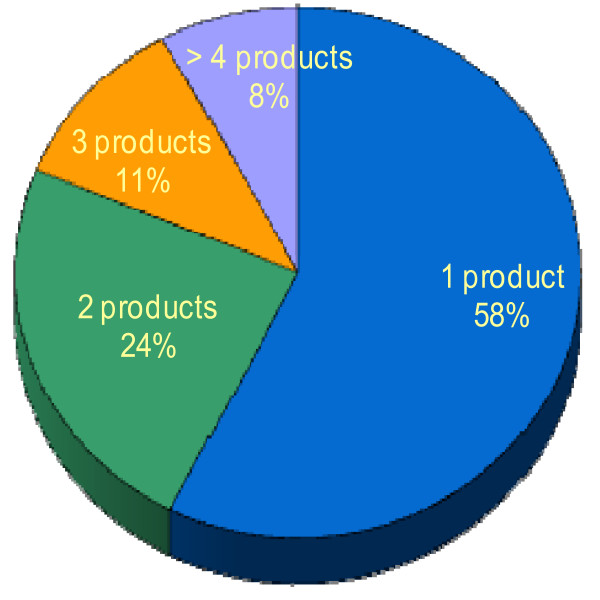
**Number of natural products taken per patient**.

Of the 104 patients reporting natural product use, the majority reported using only 1 product (60 patients, 58%). Only 8 patients reported using more than 4 natural products (Figure 1).

**Figure 2:**
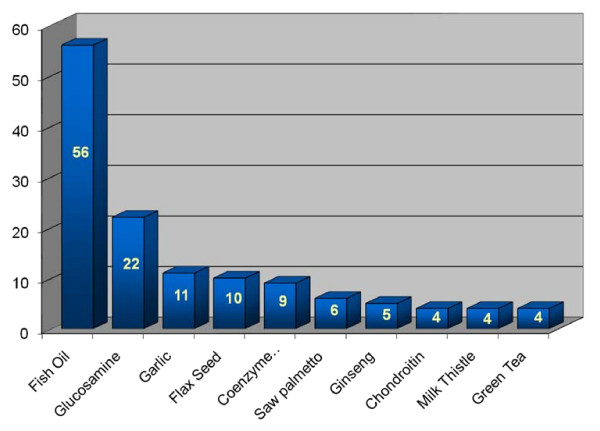
**Top 10 natural products used by surgery patients**.

When evaluating the type of natural products used by patients, fish oil was the most commonly reported (56 patients, 54%). Other reported natural products included glucosamine (22 patients, 21%), garlic (11 patients, 11%), flax seed (10 patients, 10%), coenzyme Q-10 (9 patients, 9%), saw palmetto (6 patients, 6%), ginseng (5 patients, 5%), chondroitin (4 patients, 4%), milk thistle (4 patients, 4%), and green tea (4 patients, 4%). As reported previously, some patients reported using more than 1 natural product (Figure 2).

## Discussion

In this retrospective review of surgery patients presenting to APEC, approximately one-fourth of patients indicated the use of natural products. Patients taking natural products were significantly older, were more likely to undergo cardiac or chest surgery, and were more likely to be taking more prescription and non-prescription medications than patients not reporting natural product use.

We believe our study to be the first study conducted to evaluate the natural product use and recommendations given prior to surgery. Other researchers have investigated natural product use among different populations but not recommendations given to patients regarding discontinuation and surgery. The study highlights the increased use of natural products among all patient populations, the perioperative concerns of natural product use, and the lack of available discontinuation guidelines for natural products.

This study has several limitations. First, the interview process is very subjective, and the interviews are mostly completed by University of Kansas School of Pharmacy experiential rotation students who are only in the APEC clinic for 1 month. Without being questioned, patients may not be forthcoming with their natural product use as explained previously, and while there is information regarding natural product discontinuation provided in 2 of the 3 patient rooms, it is incomplete and does not provide information on the majority of natural products identified in this study. Conversely, we assume that all patient reported medications were recorded on the form. We feel as if the recorder took the time to ask the patient about their natural product use, they also took the time to document such.

Additionally, other concerns include that all patients scheduled for surgery are not referred to the clinic, the clinic accepts appointments on a walk-up basis, and patients taking natural products presented to APEC approximately 6 days prior to surgery. The American Society of Anesthesiologists, while having no official statement, recommends discontinuing all natural products 2-3 week prior to elective surgery. A literature evaluation found that fish oil, glucosamine, saw palmetto, chondroitin, and milk thistle should be discontinued 2-3 weeks prior to surgery [15]. Conflicting evidence was found for garlic and ginseng with 1 source stating discontinuation should occur at least 1 week prior to surgery and another stating at least 2 weeks prior to surgery [1,2,4,10]. Without proper discontinuation and/or continued use, perioperative concerns include perioperative bleeding, cardiac side effects (hypertension, hypotension, tachycardia, angina), water/electrolyte disturbances, hypoglycemia, and prolongation of anesthetic effects (Table 3). Thus, if patients are not advised by their surgeon to discontinue natural products at least 2 weeks prior to their procedure, it may be too late to provide such a recommendation by the time the patient is seen at APEC and complications, such as perioperative bleeding, may ensue.

**Table 3:** Recommendations and implications of natural product use as related to preoperative concerns.

**Natural Product**	**Evidence based Recommendations:** **Discontinuation of natural products prior to surgery**	**Perioperative Concerns**
Fish Oil	2-3 weeks prior to surgery [15]	Perioperative bleeding [15,20] Hypotension [20]

Glucosamine	2-3 weeks prior to surgery [15]	Hypoglycemia [15,19]

Garlic	1 week [15,16] At least 2 weeks [22]	Perioperative bleeding [9,13,15-17,19-22] Hypotension [9,15,16,19,20]

Flax Seed	No specific recommendations	Perioperative bleeding [18]

Coenzyme Q-10 (ubiquinone)	No specific recommendations	Hypotension [20] Cardiac effects [20] Perioperative bleeding effects [21]

Saw palmetto	2-3 weeks prior to surgery [15]	Perioperative bleeding [9,15] Tachycardia [9] Angina [9] Water/electrolyte disturbances [19]

Ginseng	At least 7 days [13,15] At least 2 weeks [22]	Hypoglycemia [9,13,15-17,19] Hypertension [9,13,15,16] Perioperative bleeding [9,13,15,17,19-22] Tachycardia [9,13] Water/electrolyte disturbances [19] Prolongation of anesthetic effects [19]

Chondroitin	2-3 weeks prior to surgery [15]	Perioperative bleeding [15,19]

Milk Thistle	2-3 weeks prior to surgery [15]	Volume depletion [15]

Green Tea	No specific recommendations	Perioperative bleeding [18,21] Cardiovascular side-effects [18,21] Water/electrolyte disturbances [19]

While this retrospective survey did not specifically address the knowledge of physicians at KUH regarding discontinuation recommendation of natural products, it can be surmised that it is consistent with trends seen across the country. Heller, et al reported in a general population study that all physicians correctly knew the side effects of only 1 natural product (ephedra) and physicians did not recommend stopping 85% of natural products in the perioperative period.

## Conclusion

Based on the results of this study, it is concluded that there is a need for established guidelines regarding discontinuation of selected natural products prior to surgery and further education is needed concerning the perioperative implications of natural products.

## Competing interests

The authors declare that they have no competing interests.

## Authors' contributions

ARK and FSR designed the study, collected and compiled data, evaluated the literature, and drafted the manuscript. DWG performed the statistical analysis. JAG provided support and mentorship for the project. All authors read and approved the final manuscript.

## Pre-publication history

The pre-publication history for this paper can be accessed here:


